# Association between iron-folic acid supplementation and pregnancy-induced hypertension among pregnant women in public hospitals, Wolaita Sodo, Ethiopia 2021: a case- control study

**DOI:** 10.1186/s12889-023-15794-6

**Published:** 2023-05-10

**Authors:** Abiyot Wolie Asres, Serawit Samuel, Wakgari Binu Daga, Atsede Tena, Afework Alemu, Shimelash Bitew Workie, Mihiretu Alemayehu, Habtamu Messel

**Affiliations:** 1 Department of Epidemiology and Biostatistics, School of Public Health, Wolaita Sodo University, Wolaita Sodo, Ethiopia; 2 Department of Reproductive Health and Nutrition, School of Public Health, Wolaita Sodo University, Wolaita Sodo, Ethiopia; 3 School of Public Health, Wolaita Sodo University, Wolaita Sodo, Ethiopia; 4 Department of Pediatrics, School of Medicine, Wolaita Sodo University, Wolaita Sodo, Ethiopia; 5 Health Professionals Education Partnership Initiative Project Office, Addis Ababa University, Addis Ababa, Ethiopia

**Keywords:** Iron-folic-acid supplementation, Association, Pregnant women, Case–control, Ethiopia

## Abstract

**Background:**

Pregnancy-induced hypertension is the new onset of high blood pressure after 20 weeks of gestation in women with previously normal blood pressure. To the best of our knowledge, no study has been conducted in our country to investigate the association between this pregnancy problem and iron-folic acid supplementation. The aim of this study was to determine the association between iron-folic acid supplementation and pregnancy-induced hypertension (PIH) in pregnant women at public hospitals in the Wolaita Sodo zone.

**Methods:**

An institution-based case–control study was conducted among pregnant women who visited public hospitals in the Wolaita Sodo zone from March 3, 2022, to August 30, 2022. A consecutive sampling method was used to select the study participants. The total sample size was 492, of which 164 were cases and 328 were controls. The data were collected by conducting face-to-face interviews and measurements. The data were entered into EpiData version 4.6 and exported to STATA 14 for analysis. Those variables with a *p*-value less than 0.05 were considered statistically significant. Descriptive statistics and odds ratios were presented using texts, tables, and figures.

**Results:**

A total of 471 women participated in this study, yielding a response rate of 96%. The cases had a mean age of 25 ± 4.43, while the controls had a mean age of 25 ± 3.99. The mean age at first pregnancy among cases was 20 ± 2.82 and among controls was 20 ± 2.97. The average number of deliveries for cases and controls was 1.97 ± 1.41 and 1.95 ± 1.38, respectively. There is no significant association between iron-folic acid supplementation and PIH. Pregnant women with high hemoglobin levels had higher odds of PIH as compared to those without it (AOR = 3.65; 95% CI: 1.0–12.9). Eating kocho (AOR = 14.4; 95% CI: 1.2–16.7) was positively associated with PIH.

**Conclusions:**

There is no association between iron-folic acid supplementation during pregnancy and pregnancy-induced hypertension. Pregnant women with high hemoglobin levels had higher odds of PIH as compared to those without it. There is an association between kocho consumption and PIH. More research should be done using stronger designs.

## Background

Pregnancy-induced hypertension is the new onset of high blood pressure after 20 weeks of gestation in women with previously normal blood pressure [1]. According to a study conducted by the World Health Organization (WHO) from 2003–2009, the leading causes of maternal death were hemorrhage (27·1%), pregnancy-induced hypertension (14·0%) and sepsis (10·7%). These were responsible for more than half of all maternal deaths worldwide [2]. Pregnancy-induced hypertension (PIH) is the most common medical complication of pregnancy, with an incidence of between 5 and 10%. The WHO estimates that at least one woman dies every 7 min from a complication of PIH [3]. The incidence of PIH increased from 16.30 million to 18.08 million globally, with a total increase of 10.92% from 1990 to 2019 [4].

The burden of PIH is high in Africa, with one in 10 pregnancies affected. The burden is significantly higher in Central and Western Africa [5]. Sub-Saharan Africa accounted for approximately 86% of the estimated global maternal deaths in 2017 [6]. During the ten-year period (2006–2015), there was an increase in the number of maternal deaths due to direct causes of pregnancy [7].

The maternal mortality rate in Ethiopia declined from 5.51% to 4.98% from 2014 to 2017, respectively. Nevertheless, maternal mortality due to PIH has increased [8, 9]. Obstetric factors were directly responsible for 51 (86%) of all maternal deaths in Ethiopia. The primary direct causes of maternal mortality in Ethiopia were hemorrhage (45%), PIH (23%), and obstructed labor (18%). Among PIHs, preeclampsia was the most common type in the country [10–13].

Factors associated with PIH were: primigravida, extreme age, early gestational age, twin pregnancy, gravidity, long inter-pregnancy intervals, chronic hypertension, history of diabetes, multiple pregnancies, obesity, smoking, socioeconomic level, and diet [14–19]. Anemia and coffee intake during pregnancy are risk factors for the development of PIH [20]. Similarly, the level of iron was significantly higher in the PIH groups than in the control groups [21]. Antioxidant supplementation was associated with better maternal and perinatal outcomes than iron and folic acid supplementation alone [22]. The consumption of seafood was inversely associated with the odds of developing PIH [23]. PIH was less frequent in women who ate received iron and folic acid supplements [24]. Maternal ferritin concentration is primarily a reflection of maternal iron status, and a high level is associated with unfavorable outcomes. This indicates the need for further study of routine iron-folic acid supplementation in pregnant women [25].

The potential harmful effects of iron-folic acid were not carefully debated in regards to its effectiveness. Even if iron-folic acid is beneficial for neonatal or maternal outcomes, it is associated with glucose impairment and pregnancy-induced hypertension in mid-pregnancy [26]. High hemoglobin levels in women who took iron-folic acid supplements were associated with an increased risk of PIH [27]. Its supplementation before 16 weeks of gestational age was significantly associated with an increased risk of developing PIH [28]. But there was no association between the occurrence of PIH and the timing of iron-folic acid (IFA) supplementation. Early (< 28 weeks) and late (≥ 28 weeks) onset or start of iron-folic acid supplementation, on the other hand, was found to be protective [29, 30]. Women in the lowest iron quartile had a 2.2-fold increase in PIH risk compared to women in the highest quartile [31]. In India, women who had a diet that was sufficiently varied and supplemented with iron and folic acid throughout pregnancy experienced fewer PIH symptoms. PIH symptoms were 36% lower in mothers who took iron-folic acid supplements for at least 90 days during their previous pregnancy [32].

A study showed that serum ferritin and serum iron were higher in PIH women [33]. The serum iron level has a direct correlation with the level of blood pressure, concentration of total hemoglobin, and serum iron [34, 35]. Iron supplementation during pregnancy may have resulted in iron overload, which may have resulted in oxidative stress and endothelial dysfunction in the patients [36]. In contrast, there was no significant difference between PIH and serum iron concentrations [37].

PIH can be avoided through early detection and eating vegetables and fruits during pregnancy. The WHO highly suggests that pregnant women take daily oral iron and folic acid supplements to prevent maternal anemia, puerperal sepsis, low birth weight, and premature birth. Iron-folic acid should be started as soon as possible to prevent neural tube defects [38]. It was found that iron-folic acid supplementation is recommended for the benefit of the fetus and herself, but the prevalence of PIH is increasing, unlike other complications during pregnancy. Most factors in PIH were assessed using cross-sectional studies, which could not show a cause-and-effect relationship. Hence, the association between iron-folic acid supplementation and pregnancy-induced hypertension is not clear yet. Furthermore, to the best of our knowledge, there is no study done in Ethiopia on this association. Therefore, the aim of this study was to determine the association between iron-folic acid supplementation and pregnancy-induced hypertension among pregnant women.

## Methods

### Study design and settings

The aim of this study was to determine the association between iron-folic acid supplementation and pregnancy-induced hypertension among pregnant women in the public hospitals of Wolaita Sodo zone. An institution-based, unmatched case–control study was conducted among pregnant women attending ANC and admitted for delivery in obstetrics and gynecology departments. The study was conducted in the four public hospitals of Wolaita Sodo zone in southern Ethiopia from March 3, 2022, to August 30, 2022. The Southern Nation Nationalities and Peoples Regional State (SNNPR) is one of the ten regions that has a wide variety of nations and nationalities with different cultures, languages, lifestyles, weather conditions, topography, habitats, and other natural phenomena. The region has 16 zones, of which the Wolaita Sodo zone is one. Wolaita Sodo town is found in the southern direction of Addis Ababa (the capital city of Ethiopia) and in the southwest direction of Hawassa, about 329 and 151 km apart, respectively.

There are about eight governmental hospitals and two private hospitals in the Wolita Sodo zone. They include Wolaita Sodo University Comprehensive Specialized Hospital (WSUCSH), Bodity, Bitena, Bele, Bombe, Gesuba, Humbo, and Kindo Halale primary hospitals. The Wolaita Sodo University College of Health Science and Medicine is located in Wolaita Sodo town, which is 151 km west of Hawassa and 329 km south of Addis Ababa.

### Case definition

Cases are defined as pregnant women whose blood pressure was greater than or equal to 140/90 mmHg in two separate readings taken 4 h apart [1]. They were diagnosed and confirmed by obstetrics and gynecology physicians. Controls are defined as pregnant women in the same hospitals whose blood pressure is less than 140/90 mmHg after 20 weeks of gestation. During the study period, cases and controls were identified through record review and after physician diagnosis in ANC clinics and obstetrics and gynecology wards. The diagnosis includes history-taking, clinical manifestations, a physical examination, and laboratory tests.

### Population

The source populations were pregnant women, both cases and controls, who attended ANC and were admitted for delivery in public hospitals in the Wolaita Sodo zone. The study population consisted of pregnant women who fulfilled the eligibility criteria. These were both cases and controls who attended ANC and were admitted for delivery in the selected hospitals during the study period. Consecutively chosen pregnant women, both cases and controls, in the selected hospitals during that study period were the sampled populations.

#### Inclusion and exclusion criteria

Women who attended ANC and were admitted for delivery and had a blood pressure readings greater than or equal to 140/90 mmHg or had a blood pressure of less than 140/90 mmHg after 20 weeks of gestation were included in this study. The study excluded severely ill pregnant women.

### Sample size determination

The sample size was calculated using OpenEpi version 2.3 statistical software by assuming a proportion of cases exposed of 13%, a minimum detectable odds ratio among controls of 2.14 [28], a case-to-control ratio of 1: 2, a significant level of 95%, and a power of 80%. The sample size was 447. With a 10% non-response rate, the total sample size was 492 people, with 164 cases and 328 controls.

### Sampling procedures

**Fig. 1:**
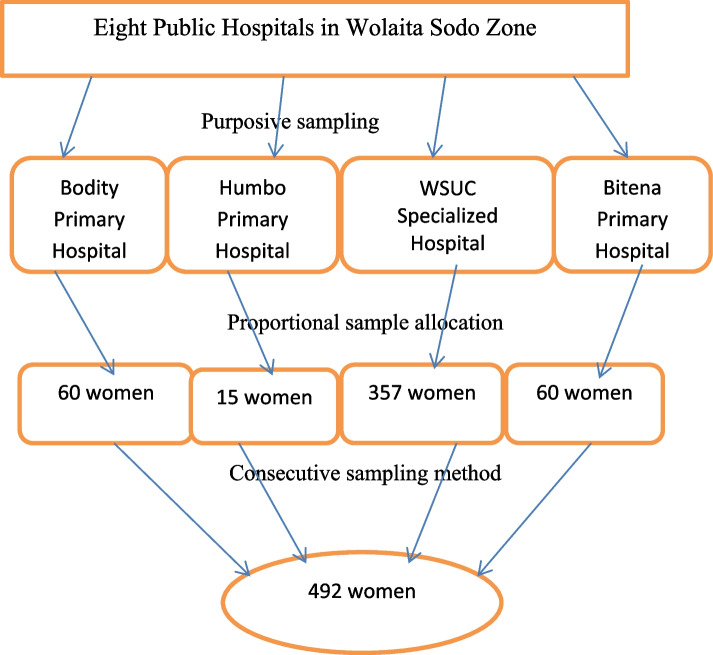
Sampling procedure to select the women in Wolaita Sodo zone public hospitals

The four hospitals—Wolaita Sodo University Comprehensive Specialty Hospital (WSUCSH), Bitena, Bodity, and Humbo Primary Hospitals—were selected due to their high patient flow rates. The cases that fulfilled the inclusion criteria were selected using the consecutive sampling method until the required sample size was attained. Then the next two immediate corresponding controls were also selected consecutively on the same day in the ANC unit and labor wards (Fig. 1).

### Instruments (Questionnaire)

The data were collected through measurements, reviewing records, and face-to-face interviews using a pretested questionnaire. The measurements included blood pressure, weight, height, and urine from the women. The women were interviewed about their socio-demographic characteristics, obstetric factors, and behavioral factors by trained and experienced health professionals immediately before and after ANC and delivery services. A questionnaire was prepared by reviewing different pieces of literature that were similar to the current study [10, 20, 24, 25, 27, 35]. Then the questionnaire was changed from English to Amharic and back-translated to English to check its consistency. The questionnaire was pretested on 5% of the sampled pregnant women.

### Laboratory measurements and individuals

Twelve health professionals—eight BSc midwives and four MPH health professionals—were recruited as data collectors and supervisors, respectively. There were two trained data collectors for each hospital. The trained laboratory technicians measured proteinuria and hemoglobin levels.

The proteinuria was measured using a dipstick test. Urine specimens for proteinuria assessment were obtained from spot urine samples collected from pregnant women who attended ANC. The health care providers used a dipstick test with a color-sensitive pad. The color changes on the dipstick indicated the women's levels of protein in the urine.

Hemoglobin levels were determined using an automated hematology analyzer machine. Blood samples were drawn from the women's veins on the inside of their elbows. Needles were inserted into the veins, and the blood samples were collected using airtight vials. The blood samples were put into the automated hematology analyzer machine. Then hemoglobin levels were determined from these blood samples using this machine. The hemoglobin levels were categorized as >  = 11.0 mg/dl and < 11.0 mg/dl [39].

### Data quality management

Training was given for data collectors and supervisors about two days before data collection. A clear explanation of the purpose of the study was provided to the respondents at the beginning of the interview. Supervisors and the principal investigator provided close supervision. The data from each respondent was checked for completeness, clarity, consistency, and accuracy by the data collectors, supervisors, and the principal investigator.

### Statistical analysis

After data collection, the data were coded and entered using Epidata version 4.6 software. The entered data was then transformed into the STATA 14 version. Descriptive statistics like frequencies, percentages, means, and standard deviations were done. The association between pregnancy-induced hypertension and each variable was checked using bivariate logistic regression. Variables with a *p*-value less than 0.25 in the bivariate logistic regression were entered into multivariable logistic analysis, and those variables with a *p*-value of less than 0.05 in multivariate logistic regression were considered statistically associated factors. Text, tables, and figures were used to present the findings.

## Results

### Socio-demographic characteristics

A total of 471 women participated in this study. The response rate was 96%. The cases had a mean age of 25 ± 4.43, while the controls had a mean age of 25 ± 3.99. The mean age at first pregnancy among cases was 20 ± 2.82 and among controls was 20 ± 2.97. The average number of deliveries for cases and controls was 1.97 ± 1.41 and 1.95 ± 1.38, respectively. About 153 (97.5%) of cases and 304 (96.8%) of controls were married. Regarding educational status, 12 (7.6%) of the cases and 11 (3.5%) of the controls could not read or write. Similarly, 79 (50.3%) of cases and 136 (43.3%) of controls were housewives. More than half (51.0%) of the cases and 198 (63.0%) of the controls were from urban residences (Table 1).

**Table 1:** Socio-demographic characteristics of the women

Variable	Response category	Women’s status	
		**Have PIH (Case)** **No (%)**	**No PIH (Control)** **No (%)**
**Age of the women**	18–20 years	29(18.5)	45(14.3)
	20–30 years	115(73.2)	246(78.4)
	30–40 years	13(8.3)	23(7.3)
**Marital status**	Single	4(2.5)	10(3.2)
	Married	153(97.5)	304(96.8)
**Educational status**	Cannot read and write	12(7.6)	11(3.5)
	Can read and write	145(92.4)	303(96.5)
**Occupation of the women**	Housewife	79(50.3)	136(43.3)
	Merchant	27 (17.2)	71(22.6)
	Government employee	32 (20.4)	78(24.8)
	Other	19(12.1)	29(9.3)
**Residence**	Urban	80(51.0)	198(63.0)
	Rural	77(49.0)	116(37.0)

### Obstetric and gynecological factors

About 100 (63.7%) of the cases and 225 (71.7%) of the controls were multigravida, and 9 (5.7%) of the cases and 11 (3.5%) controls had multiple pregnancies (Table 2).

**Table 2:** Obstetrics and gynecological characteristics of the women

Variables	Response category	Women status	
		**PIH No (%)**	**No PIH No (%)**
**Age at first pregnancy**	12–20 years	103(65.6)	228(72.6)
	20–30 years	54(31.4)	86(27.4)
**Gravidity (number of pregnancies)**	Primigravida	57(36.3)	89(28.3)
	Multigravida	100(63.7)	225(71.7)
**Parity (number of deliveries)**	Nulipara	80(51.0)	155(49.4)
	Para > = 1	77(49.0)	159(50.6)
**Interval of pregnancies**	< = 2 years	65(67.7)	142(64.3)
	> 2 years	31(32.3)	79(35.7)
**Number of fetus in current pregnancy**	Single	148(94.3)	303(96.5)
	Multiple	9(5.7)	11(3.5)
**Did you attend antenatal care?**	Yes	147(93.6)	293(93.3)
	No	10(6.4)	21(6.7)

### Iron-folic acid supplementation related factors

One hundred thirty (82.8%) of the cases and 252 (80.2%) of the controls had taken iron during their pregnancies. After 16 weeks of gestation, 113 (86.9%) of the cases and 228 (90.5%) of the controls began their first dose of iron and folic acid supplementation. In terms of hemoglobin levels, 115 (73.2%) cases and 211 (67.2%) controls had levels greater than or equal to 11.0 g/dl. Only 42 (26.8%) cases and 103 (32.8%) controls had hemoglobin levels below the normal range (Table 3).

**Table 3:** Iron folic-acid supplementation related characteristics of the women

Variable	Response category	Women status	
		**PIH No (%)**	**No PIH No (%)**
**Did you take iron-folic acid during your ANC follow-up?**	Yes	130(82.8)	252(80.2)
	No	27(17.2)	62(19.8)
**At what GA did you start?**	Before 16 weeks	17(13.1)	24(9.5)
	After 16 weeks	113(86.9)	228(90.5)
**Had you got counseling about the importance of taking the pills?**	Yes	129(99.2)	249(98.8)
	No	1(0.8)	3(1.2)
**How did you take the tablet per day**	One pill per day	121(93.1)	229(90.9)
	Two pills per day	6(4.6)	15(5.9)
	Three or more per day	0(0.0)	1(0.4)
	Sometimes	2(1.5)	6(2.4)
	Not remembered	1(0.8)	1(0.4)
**Did you take the pills correctly?**	Yes	80(61.5)	171(67.8)
	Withdraw	46(35.4)	71(28.2)
	Not start	4(3.1)	10(4.0)
**If no what was or were the reason/s?**	Fear of side effect	17(36.9)	25(30.5)
	Forgetfulness	9(19.6)	29(35.4)
	Unpleasant test	9(19.6)	10(12.2)
	Excessiveness	7(15.2)	8(9.7)
	Others	4(8.7)	10(12.2)
**What is the importance of iron for the mother?**	To prevent anemia	75(47.8)	168(53.5)
	To prevent the fetus from danger	57(36.3) 8(5.1)	74(23.6) 32(10.2)
	Other	1(0.6)	3(0.9)
	Do not know	16(10.2)	37(11.8)
**Hemoglobin label**	< 11.0 g/dl	42(26.8)	103(32.8)
	> = 11.0 g/dl	115(73.2)	211(67.2)
**Did you told that you have anemia?**	Yes	12(7.6)	25(8.0)
	No	145(92.4)	289(92.0)
**Have you ever had history of induced abortion?**	Yes	29(18.5)	45(14.3)
	No	128(81.5)	269(85.7)
**If yes what was number of abortion?**	1	16(55.2)	37(82.2)
	> = 2	13(44.8)	8(17.8)
**Have you ever used contraceptive method before the current pregnancy?**	Yes	104(66.2)	223(71.1)
	No	53(33.8)	91(28.9)

### Behavioral factors

We have also assessed the behavioral and nutritional histories of the women who consumed during their pregnancies. Most of these nutrients are sources of iron. Among the commonly known sources of iron are teff (Eragrostis tef), animal products, cereals, and fish. Based on this study, 45 (28.7%) of cases and 112 (35.6%) of the controls ate injera that was prepared from teff [40]. Similarly, 78 (49.7%) of cases and 138 (43.9%) of controls consumed sweet foods or soft drinks [41] during this pregnancy (Table 4).

**Table 4:** Behavioral factors of the women

Variable	Category	Women status	
		**PIH No (%)**	**No PIH No (%)**
Have you ever drunk alcohol during current pregnancy?	Yes	17(10.8)	34(10.8)
	No	140(89.2)	280(89.2)
Have you ever drunk coffee during current pregnancy?	Yes	124(78.9)	236(75.2)
	No	33(21.1)	78(24.8)
Have you ever drunk Chemo during current pregnancy?	Yes	137(87.3)	245(78.1)
	No	20(12.7)	69(21.9)
If yes, what was the frequency of chemo drank?	> = 2 times/day	14(10.3)	16(6.6)
	Everyday	20(14.7)	39(15.9)
	5–6 times/week	11(8.1)	10(4.1)
	3–4 times/week	29(21.3)	49(20.1)
	1–2 times/week	52(38.2)	112(45.9)
	1–3 times/month	10(7.4)	18(7.4)
Have you ever eating vegetables during this pregnancy?	Yes	150(95.5)	306(97.5)
	No	7(4.5)	8(2.5)
Have you ever eating fruit during this pregnancy?	Yes	151(96.2)	307(97.8)
	No	6(3.8)	7(2.2)
Have you ever eating kocho/bulla during this pregnancy?	Yes	136(86.6)	242(77.1)
	No	21(13.4)	72(22.9)
From which of the following you prepared injera?	Teff	45(28.7)	112(35.7)
	Maize	8(5.1)	16(5.1)
	Both	104(66.2)	186(59.2)
Have you ever been eating fish during this pregnancy?	Yes	47(29.9)	113(30.9)
	No	110(70.1)	201(69.1)
Have you ever been eating animal product during this pregnancy?	Yes	147(93.6)	309(98.4)
	No	10(6.4)	5(1.6)
Have you ever eating cereals during this pregnancy?	Yes	149(94.9)	306(97.4)
	No	8(5.1)	8(2.6)
Have you ever eating sweet foods or soft drinks during this pregnancy?	Yes	78(49.7)	138(43.9)
	No	79(50.3)	176(56.1)

### Association of iron supplementation and PIH

We used logistic regression analysis to identify factors associated with pregnancy-induced hypertension. Based on this, about nine variables were eligible for multivariable logistic regression analysis. There is no association between iron-folic acid supplementation and PIH. Even if our main objective was to determine the association between iron-folic acid supplementation and pregnancy-induced hypertension, we also assessed other dietary factors that could be sources of iron. Hence, hemoglobin levels and the consumption of kocho or bulla were found to be significantly associated with pregnancy-induced hypertension. The odds of PIH was 3.65 (1.0–12.9) times higher among women with a hemoglobin level >  = 11.0 mg/dl as compared with women whose hemoglobin level was less than 11.0 mg/dl (Table 5).

**Table 5:** Bivariate and multivariable analysis results of the study

Variable	Category	Women status		COR	*P*-value	AOR with 95% CI
		**PIH No (%)**	**No PIH No (%)**			
**Hemoglobin level**	< 11.0	42(26.8)	103(32.8)	1		1
	> = 11.0	115(73.2)	211(61.4)	1.34	0.196	**3.65(1.0–12.9)**
**Number of abortion**	1	16(55.2)	37(82.2)	1		1
	> = 2	13(44.8)	8(17.8)	3.76	0.014	3.2(0.9–11.2)
**Have you ever drunk chemo during your pregnancy?**	Yes	137(87.3)	245(78.1)	1.93	0.017	1.3(0.2–8.2)
	No	20(12.7)	69(21.9)	1		1
**Have you ever eaten kocho or bulla during your pregnancy?**	Yes	136(86.6)	242(77.1)	1.92	0.015	**14.4(1.2-16.7)**
	No	21(13.4)	72(22.9)	1		
**From which of the following you prepared injera?**	Teff	45(28.7)	112(35.7)	0.72	0.123	1.0(0.2–4.7)
	Maize	8(5.1)		0.89	0.802	0.2(0.01–2.3)
	Both	104(66.2)	16(5.1) 186(59.2)	1	1	1
**Have you ever eaten fish during your pregnancy?**	Yes	47(29.9)	113(35.9)	0.76	0.207	0.3(0.1–1.1)
	No	110(70.1)	201(64.1)			1
**Have you ever animal product during your pregnancy?**	Yes	147(93.6)	309(98.4)	0.24	0.010	0.2(0.0–7.7)
	No	10(6.4)	5(1.6)	1		1
**Have you ever eaten cereal during your pregnancy?**	Yes	149(94.9)	306(97.5)	0.48	0.158	0.7(0.1–7.9)
	No	8(5.1)	8(2.5)	1		1
**Have you ever eaten sweet foods drinks during your pregnancy?**	Yes	78(49.7)	138(43.9)	1.26	0.240	3.2(0.8–12.3)
	No	79(50.3)	176(56.1)	1		

## Discussion

Pregnant women with high hemoglobin levels had higher odds of pregnancy-induced hypertension as compared to controls. The finding of this study was in line with a study conducted in Iran [27]. According to the study conducted in Iran, a high hemoglobin level in the first trimester was a risk factor for pregnancy-induced hypertension. The current finding is also consistent with the study conducted in Arba Minch and Abbottabad [34, 42]. Hemoglobin determines the viscosity of blood. According to various studies, systolic and diastolic blood pressure both increase as hemoglobin levels rise [43, 44]. An increase in free hemoglobin contents results in vasoconstriction, which leads to the development of PIH [45]. In pregnancy-induced hypertension, a drop in intravascular volume and a rise in tissue edema were caused by the loss of serum protein and an increase in capillary endothelial permeability [46]. Any organ, including the liver, brain, and lungs, could be affected. The blood volume reduction can cause the maternal hemoglobin concentration to rise [47]. However, the current finding contradicts a study conducted in India [32]. The possible explanation might be that the sample size in the Indian study was very small, which might have had a small effect. In addition to this, the study design was cross-sectional as compared with the current study, which did not show the cause-effect relationship as a case–control study. Furthermore, the study populations were different between the two studies. The current study's population included all pregnant women of any age and gestational age. It is found that pregnant women who later develop PIH have considerably higher levels of hemoglobin, hematocrit, serum iron, serum ferritin, and transferrin saturation [48]. In the PIH group, the platelet indices were lower, and the serum iron levels were higher [49].

In this study, there was no association between pregnancy-induced hypertension and iron-folic acid supplementation. It is consistent with a Thailand study, which found that taking iron and folic acid supplements late in pregnancy has no effect on pregnancy-induced hypertension. But, according to this study, early initiation of iron-folic acid supplementation before 16 weeks of pregnancy dramatically raised the risk of developing PIH [27]. This variation may be due to the fact that the time of initiation was early in pregnancy, whereas in the current study we assessed the association among pregnant women irrespective of gestational age or anemia status. Similarly, women who use high-dose folic acid supplements before pregnancy and through mid-pregnancy may be at increased risk for high blood pressure [50]. This finding is contrary to a study conducted in Poland. According to that study, PIH risk was 2.19 times higher in women in the lowest iron quartile (801.20 g/L) compared to those in the highest (> 1211.75 g/L) iron quartile [31]. The rise in blood iron observed in patients with PIH appears to be caused by a persistent, clinically undetectable hemolytic response. In addition to this, supplementing women with folic acid and multivitamins that contain folic acid rather than folic acid alone throughout pregnancy considerably lowers the incidence of PIH [29, 30]. The possible reasons might be due to the differences between the study designs.

We have also assessed the association between pregnancy-induced hypertension and other nutritional-related factors. Based on this, there was another nutrient that was assessed in this study called kocho or bulla, which is one of the well-known and commonly consumed cultural foods in the study area. According to previous studies, iron is one of its constituents. There is a strong association between kocho or bulla consumption and PIH. Pregnant women who consumed bulla or kocho were 14.4 (1.2–16.7) times more likely to develop pregnancy-induced hypertension as compared with control groups [51]. This result seems to recommend that pregnant women avoid using bulla or kocho during their pregnancy, but this is the first study assessing this food and PIH. We were unable to obtain studies on the preceding or their mechanisms of action. We could not get any justification from previous literature about the effects of kocho or bulla on pregnancy-induced hypertension. Hence, it needs further study with regard to this association. We have also assessed nutrients that are rich in iron, like teff, but they had no association with pregnancy-induced hypertension.

The strength of this study is that, to the best of our knowledge, it is the first study of its kind in our country. But this study did not differentiate between the specific type of pregnancy-induced hypertension and its association with iron-folic acid supplementation. As a limitation, there might be respondents' recall biases regarding the number of iron and folic acid tablets they took, the total number of weeks they took the tablets, and the starting time of tablet taking. Some women might also hide the truth about whether they took the tablets completely or left them after taking them from the health institutions. This intern might affect the true association.

## Conclusions

There is no association between iron-folic acid supplementation and pregnancy-induced hypertension. However, pregnant women with high hemoglobin levels had a higher risk of developing pregnancy-induced hypertension than those who did not. The concentration of iron-folic acid has a direct relationship with hemoglobin levels. Before giving iron and folic acid supplements to pregnant women, it is crucial to assess their iron status because they may have more detrimental consequences than good ones. All pregnant women should have their hemoglobin levels measured as a routine task during their first visit. Strong designs like randomized clinical trials or meta-analyses should be carried out with a large sample size.

## Data Availability

The datasets used and/or analysed during the current study are available from the corresponding author on reasonable request.

## Abbreviations

ANC: Antenatal Care
AOR: Adjusted Odds Ratio
BMI: Body Mass Index
BP: Blood Pressure
DBP: Diastolic Blood Pressure
DM: Diabetes Mellitus
EDHS: Ethiopian Demographic and Health Statistics
FA: Folic Acid
GA: Gestational Age
HDP: Hypertension Disorder of Pregnancy
HEPI: Health Professionals Education Partnership Initiative
HTN: Hypertension
IFA: Iron Folic Acid
MNM: Multiple Micronutrients
OR: Odds Ratio
PIH: Pregnancy Induced Hypertension
SBP: Systolic Blood Pressure
SNNP: Southern Nation Nationalities
WSUTCSH: Wolaita Sodo University Teaching and Comprehensive Specialized Hospital
